# Building menstrual health and hygiene-supportive environments: exploring teachers’ experience in rural Western Kenya

**DOI:** 10.3389/fpubh.2023.1206069

**Published:** 2023-07-25

**Authors:** Julia L. Shenkman, Leah C. Neubauer, Linda Mason, Kelvin Oruko, Kelly Alexander, Penelope A. Phillips-Howard, Elizabeth Nyothach

**Affiliations:** ^1^Division of Public Health Practice, Department of Preventive Medicine, Feinberg School of Medicine, Northwestern University, Chicago, IL, United States; ^2^Department of Clinical Sciences, Liverpool School of Tropical Medicine, Liverpool, United Kingdom; ^3^Centre for Global Health Research, Kenya Medical Research Institute, Kisumu, Kenya; ^4^Kenya Medical Training College, Nairobi, Kenya; ^5^Water + Team, Care International, Atlanta, GA, United States

**Keywords:** school health, global health, menstrual education, menstrual products, teacher perspectives

## Abstract

**Introduction:**

Existing school environments and staff play a critical role in Menstrual Health and Hygiene (MHH) for school aged girls in middle and low-income countries. This paper leverages teachers’ perspectives on menstruation and the impact of the Menstrual Solutions (MS) study, an open cluster randomized controlled feasibility study to determine the impact of puberty education, nurses support, and menstrual product provision on girls’ academic performance and emotional well-being.

**Methods:**

Seventeen focus group discussions were conducted from October 2012 through November 2013 with teachers at six participating schools, held at three different time points during the study period.

**Results:**

Key themes that emerged were emotions and blood, absenteeism, the role of teachers in MHH, and the impact of sensitization. Teachers noted that poor MHH had an impact on school attendance, transparency and openness with teachers, and student behavior in class. It was reported that adolescent girls would absent themselves for 3–5 days during their menstrual cycle depending on what materials they could use, and they would often shy away from teachers, when possible, only speaking to them about their menses if it was urgent or they needed to go home. Emotions such as fear and embarrassment were commonly associated with bleeding. At the midpoint and end of the study, teachers noted that the puberty education and menstrual product provision (where applicable) had a positive impact on girls’ attendance, attention, and comfort in the classroom. Girls became more open with both male and female teachers about their menses, and more comfortable and confident in the classroom among all classmates.

**Discussion:**

This research highlights the importance of building an MHH-supportive environment with multiple school personnel within schools to develop a gender-equitable environment for girls to learn confidently without undue interference. Teachers are key adults in adolescent girls’ lives, having the potential to foster an environment that empowers girls with greater autonomy to manage their menses. This highlights a need to consider their perspectives in intervention development. Sensitization of teachers and puberty education across both genders are key components to developing the MHH-supportive environment in schools.

## 1. Introduction

Adequate menstrual hygiene and health (MHH) remains difficult to attain for adolescent girls in low-and middle-income countries (LMICs). According to Sommer et al. (1), girls have difficulty managing menses due to a lack of materials, facilities, and guidance. Oftentimes, girls are faced with lack of access to sanitary pads or other menstrual products and are forced to use everyday items such as cloth, mattress pieces, or blankets, with some of these supplies not being properly sanitized before use (2). Girls in previous studies reported that a lack of access to clean water and soap exacerbate this issue, preventing them from bathing or keeping their menstrual products clean (2, 3). These reported barriers are compounded by the association of menses with sex and maturity and social norms of needing to keep it a secret, a difficult task when girls do not have the necessary products to manage bleeding (4–6). With limited knowledge and guidance on menstruation prior to their first menstrual cycle, girls in LMICs are faced with the difficult task of learning from female relatives, peers, teachers, or on their own, to maintain this secrecy (5). As a result of this lack of preparedness and social concern, girls have reported feelings of discomfort, embarrassment, and fear in relation to their menstrual cycle in addition to existing stress in their daily lives (4). The literature suggests that teachers, while a potential source of information given the role that schools play in health, are the least common source of information on menstrual hygiene for adolescent girls (2, 5). Previous studies have suggested a variety of reasons for why teachers may avoid these discussions entirely, including: feeling unprepared to address this gap in information as they lack the training to do so, the cultural taboo associated with menstruation, and male teachers are uncomfortable speaking about menstruation (2, 4, 5).

During menstruation, girls report they stay home from school due to the pain from cramping, or the fear and anxiety of soiling their clothing in public (3, 7). A systematic review of sixty-four studies conducted in India reported that one in four girls (25%) missed one or more school days during their menses (6). A cross-sectional survey of schoolgirls in rural north Ghana found similar results, with a prevalence of menstruation-related absenteeism at 27.5% (8). Additional reasons for staying home from school that were cited include physical discomfort or pain, lack of hygiene materials or facilities, and critical feedback restrictions from parents or teachers (7). Missing time in school results in lost lessons and examinations that girls have to make up (8). Continued absences from class can result in repeating the year and contribute to dropping out of school (8). Even while in class, teachers have reported that girls are often distracted by their period, visibly waiting until fellow classmates leave and are absent from school after this (2). This ‘distraction’ prevents girls from concentrating in class, resulting in them missing some of the material that was taught (3). During their menses girls may go home during lunch to change or wash and may not come back; this causes an interrupted school day, resulting in missed class while potentially being marked in attendance in school records (3).

MHH has a significant impact on educational outcomes, sparking global recognition of it as an important issue in international development. MHH has been identified as a global priority by the World Health Organization (9). This initiative recognizes that schools provide an opportunity to engage children and adolescents in the promotion of healthy lifestyles (9). As such, it is important to consider MHH in the larger context of school health when developing initiatives.

Schools and teachers play an important role in how girls navigate menstruation during adolescence. A feasibility study (Menstrual Solutions Study) was conducted in primary schools in western Kenya (10). The time spent in school and the clear gap in utilization of teachers as a potential source of information about MHH underscores the need for a further evaluation of teacher perception, opinions of their roles, and knowledge of menstruation. This paper presents the findings from focus group discussions with teachers. The discussions examine teachers’ knowledge and perceptions of menstruation and evaluate its effect on girls’ education. The majority of focus group discussions and interviews conducted in LMICs on MHH are conducted with girls and their parents.

## 2. Methods

### 2.1. Study area and population

The study took place in Gem, Siaya County District in western Kenya, within KEMRI’s Health and Demographic Surveillance System (HDSS) site. The population primarily consists of subsistence farmers and fishers, the majority of whom are members of the Luo ethnic group who were attending secondary school (11).

### 2.2. Menstrual solutions study

The Menstrual Solutions (MS) study, an open cluster randomized controlled feasibility study, assessed the cultural acceptance, use, satisfaction, costs and safety of menstrual cups and sanitary pads among primary school girls in rural western Kenya (10). Primary schools were selected in the designated sub-county based on reaching a minimum threshold of water, sanitation, and hygiene (WASH), and agreement of the head teacher to participate. All schools selected were randomly assigned to one of three study arms. The first arm supplied menstrual cups to all eligible study girls in the 10 “cup” allocated schools. The second arm supplied monthly sanitary pads to all eligible enrolled girls in the 10 “pad” allocated schools. Eligible girls in the 10 “control” allocated schools in the third arm received no additional supplies and continued with their usual menstrual practice. Menstrual products for the first and second study arm were supplied through the grant funding the MS Study. Schoolgirls aged 14–16 attending those schools were eligible to participate if they had no precluding disability, experienced at least three menstrual periods, and were residents in the study area for at least 4 months (10). In addition, the girls in the study were provided with soap each term and received puberty education at the start of the study. Monthly surveys were conducted using self-completed netbooks, and interviews with study nurses. Results from this study, in addition to a detailed description of the three study arms are described in Phillips-Howard et al. (10). The study was approved by the KEMRI Scientific and Ethical Review Board (SSC No 2198), and the Institutional Review Boards of the Liverpool School of Tropical Medicine (11, 12).

### 2.3. Teacher focus group recruitment

The research team randomly selected 6 schools out of the 30 schools enrolled in the MS study to conduct FGD, with 2 schools from each of the three study arms to conduct FGD with both teachers and adolescent girls. The results from the FGD with adolescent girls are reported in Mason et al. (3, 13).

FGD were conducted at three different time points, Baseline, Midline, and Endline, to gage the impact of the interventions, and the changes in teacher’s perspectives given these interventions. One school was unable to complete a baseline FGD (School 6), resulting in a total of 17 FGDs with teachers. All FGD were completed from October of 2012 through November of 2013.

Participant teachers were recruited at school level, with all teachers invited to join a school-specific FGD. The qualitative team visited the school and invited all teachers within the school to participate. Teachers available on the day of the FGD attended. No teacher declined or withdrew from the study. Written consent was obtained from all teachers that volunteered to take part in the FGDs. Drinks (soda) and snacks were provided during the FGD, in addition to a reimbursement of Ksh 400 (approximately $4.00 USD at the time). This represents an average equivalent of a fare one would use to and from the school.

### 2.4. Focus group discussion approach

Semi-structured FGD guides were developed to facilitate dialogue and understanding of key issues across groups. The topics discussed in the FGD mirrored those that were discussed in the student FGD. Topics focused on: *cultural attitudes, menstrual management techniques, impact on education, the role of teachers in assisting adolescent girls, school curriculum on menstruation, the impact of sensitization, and use of the menstrual cup.* Focus groups were conducted in a mix of English and Luo by a moderator with the assistance of a note taker. Both the moderator and the note taker were trained in qualitative methods, staff of KEMRI, and were residing outside of the study area. As a result, participants were unlikely to know the interviewer or note taker outside the study context. They were young local Luo females that were fluent in both Luo and English. Also present at the discussions was a senior member of the research team. Discussions lasted between 1 and 2 h, including introductions, ground rules, and informed consent. All teachers were provided with an information sheet ahead of the FGD to facilitate an efficient consent process. These were digitally recorded after approval was given by participants. Notes were taken to capture key points, group dynamics, and non-verbal gestures. All teachers that participated were assigned a number for identification in the transcript and no names were taken. The recordings were transcribed verbatim and then translated from Luo to English by a trained KEMRI research staff member that did not participate in *that* FGD. Ongoing review of the data were conducted during the FGD process to ensure data saturation. Redundancies in the data confirmed the topics and emerging themes.

### 2.5. Analysis

The data from all 17 FGD were analyzed using thematic content analysis (14). Following translation and back translation into English, the English transcripts were read several times by the first and second author who were part of a team of 4 researchers. These two worked to develop an initial coding frame based on the key themes that emerged. The transcripts were then coded using Dedoose by the first author based on the coding frame, with additional codes added as new themes emerged. Sub-codes were developed after the initial coding, with transcripts being recorded as necessary based on these additions. The completed codebook and quotes were reviewed by the third and sixth author to verify consistency, interpretation, and add codes and subcodes.

The first section of the results focuses on the Baseline FGD and identifies how girls manage their menses. The remaining sections on absenteeism, the role of teachers in MHH, and sensitization highlight changes in results from baseline to midline and endline on those topics.

## 3. Results

In total 169 teachers among 17 FGD, an average of 10 teachers per FGD (Table 1) across the baseline, midline and endline. A total of 95 teachers attended at least one FGD, with 74 multiple attendances, resulting in 169 attendances of teachers across all FGD. The teachers at each point in time were not all the same, as some teachers had changed schools or were not available for each of the discussions. The overall demographics of the participants can be found in Table 2. Both male and female teachers participated in the FGD, with the majority of participants being male (60%). A total of 73% of the teachers were married. Length of time teaching ranged from 2 months to 33 years, with the average being 13.4 years.

**Table 1:** Focus group discussion breakdown.

Timepoint	School	Duration	Participants	Intervention
Baseline	1	90 min	5	Mooncup
Baseline	2	100 min	12	Pads
Baseline	3	105 min	12	Usual
Baseline	4	135 min	10	Usual
Baseline	5	138 min	7	Mooncup
Midline	1	150 min	8	Mooncup
Midline	2	82 min	12	Pads
Midline	3	84 min	8	Usual
Midline	4	78 min	12	Usual
Midline	5	110 min	12	Mooncup
Midline	6	121 min	12	Pads
Endline	1	93 min	5	Mooncup
Endline	2	95 min	12	Pads
Endline	3	95 min	12	Usual
Endline	4	85 min	11	Usual
Endline	5	107 min	10	Mooncup
Endline	6	93 min	9	Pads

**Table 2:** Participant demographics.

Characteristic	*n* (%); *N* = 95
Gender	
Male	57 (60)
Female	38 (40)
Marital status	
Married	69 (73)
Single	26 (27)
Ethnicity	
Luo	93 (98)
Luhya	1 (1)
Kalenjin	1 (1)
Length teaching	
≤ 10 years	54 (57)
11–20 years	10 (11)
21–30 years	25 (26)
≥ 31 years	6 (6)
Range	2 months – 33
Mean (years)	years 13.4
Mean Age (years)	37

The teachers primarily spoke of their personal experiences about (non-identified) students in their classes, which informed the following major emerging themes: management of menstruation at baseline, concerns of absenteeism, the role of teachers in MHH, and the impact of sensitization.

### 3.1. Management of menstruation at baseline

Teachers considered that the sight and smell of blood is a clear indicator of a girl being on her menses. When teachers or other classmates noticed stains or smelled the blood, some teachers noted that the girls reacted poorly to it, making the girls feel self-conscious and shy about their menses. The majority of teachers reported that despite significant attempts at privacy, girls were described as being ill-equipped to manage their menses, and inevitably faced ridicule from others around them. As one teacher noted, *“this fear comes about because the girl knows very well that she is not protected and whenever she is going to stand, back there is going to be stained, and you can imagine that embarrassment with the boys and so on*” (P4 School 3, Baseline). Another teacher noted that some girls will attempt to hide these stains to avoid embarrassment.

*“Sometimes they come to ask for the permission they tie the pullover around the waist, you find that maybe she has stained the clothe, some of them take chalk when they have stained the clothe, they decolor it, the stain to hide the stain, so you can think that that is a chalk stain, not blood stain”* (Px School 4, Baseline).

These experiences of teasing can be detrimental to girls’ emotional state and academics, with one teacher highlighting the experience of a girl who dropped out due to the teasing from her classmates. As they noted, *“the other pupils were laughing and the dress was like wet, after that, okay she went home and she did not come back, she went for good”* (P6 School 5, Baseline).

To address menses at school, some girls will ask to go home and change because they do not have the materials they need at school. *“You see she comes and asks for permission, goes and changes then she comes back”* (Px, School 3, Baseline). Some would also change at home due to inadequate facilities at school, with one teacher stating, *“I know in the toilet there is not going to be privacy because it is a place where very many go”* (P6 School 3, Baseline). This was not the case at all schools however, with other teachers at different schools noting that the facilities were adequate. As one teacher stated at their school *“we simply take the girl to the wash room, we simple tell the girl to take water in the wash room and we provide soap that we have in the school and the girl takes bath”* (P5 School 1, Baseline).

Other girls missed school to avoid a leak (as discussed in the next section).

### 3.2. Concerns of absenteeism

Teachers across all study schools noted issues of absenteeism of girls during their menstrual cycle. Girls would tell their teachers they were sick, without providing any details when leaving or returning from school: “*they will say I was sick, they will not say it [they are on menses] openly; they will just say I was sick and that is all”* (P4 School 4, Baseline). All teachers reported at baseline that some girls were not comfortable with speaking with their teachers at all. It was reported that girls would send some of their friends, *“just go and tell mwalimu [teacher] I am very sick, so they will come and say so and so is sick and would like to go home”* (P2 School 5, Baseline).

They identified three key reasons why girls would be absent from school while on their menses. The first was fear of their classmates, noting that they were experiencing their menses. “*Some fear to be noticed by their fellows that they are on their periods”* (P9 School 3, Baseline). The second reason was attributed to not having access to adequate menstrual supplies due to low socioeconomic status. As stated by one teacher, *“most of these kids they do come from poor families and their parents cannot afford to buy them pads, so they will take even more than 3 days until their periods are over”* (P4 School 5, Baseline). The third reason teachers cited was menstrual cramps. They recognized that some of the girls “*have severe stomachache [cramps and] hence cannot go to school*” (P9 School 3, Baseline).

Teachers noted that absences varied depending on the girls’ situation at home, stating that,

*“They take time off school especially at puberty some girls comes from homes that parents are enlightened and so they are able to manage their menses and some are not able to manage their menses because they are coming from families that are not enlightened”* (P1 School 2, Baseline).

Loss of time at school resulted in missing curriculum that had to be made up. As one teacher noted, *“Of course yes, whatever concept they missed during the normal learning process actually we bring this and tell them that they need to accomplish what other pupils had done in their absence”* (P5 School 1, Baseline). It was reported that some schools are not equipped to handle the absences due to a large number of students per teacher, which they acutely recognized: *“Of course there should be something done like maybe remedial teaching, but you see with the current staffing that… may be some go like that undetected”* (P6 School 1, Midline). Teachers that noted this trend said schools were unable to provide individual support to girls that miss lessons due to menstrual issues. “*You cannot give attention to one pupil because she was not there, so you just have to go on with the lessons, so in case she was not around she just have to consult with the others*” (P1 School 3, Midline).

Most teachers noted that the presence of blood can force girls to leave school out of either fear or necessity to change, impacting time in class. Once they are seen with a blood stain and teased for it, they are more likely to stay home for the duration of their menses. One teacher noted *“when these unexpected period comes the child becomes too shy because the pupils will be laughing at her so when the child goes home with that shame she will say no, I am not going back”* (P11 School 5, Midline). Some teachers stated they would sometimes send girls home to change as well. *“If a girl had started leaking which happens rarely, I would give her chance to evacuate the classroom and advize her that maybe, she goes home and do the necessary thing”* (P7 School 6, Midline).

With the puberty education intervention, teachers at each school saw improvement in absenteeism midway and at the end of the study. *“Absenteeism is totally minimized, they have been coming to school regularly and in fact they are neat nowaday”* (P4 School 2, Midline). While absences related to menstruation were not eliminated, most teachers noted that *“if you look at the frequency, it has reduced”* (P6 School 4, Midline). Those who previously had been absent were described as better able to communicate with teachers about their menstrual problems. “*Earlier on they never used to [tell teachers about their menses], but these days they are very courageous. They approach us and tell us why they were absent”* (P8 School 6, Midline).

In discussions at midline and endline, several teachers reported a positive impact on academic performance. *‘The girls are always there, just like the boys have been attending regularly, so this result in the performance being better’* (P12 School 2, Midline). Relating this back to unequal instruction of boys and girls, one teacher stated, *“this question of boys getting more learning hours per week per month per term is now a thing of the past”* (P8 School 6, Endline).

### 3.3. The role of teachers in menstrual health and hygiene

In the focus group discussions, the teachers discussed the role that teachers play in menstruation, since girls spend a significant amount of time at school in their presence. Some noted that they do not feel prepared to address girls’ concerns or questions about their menses while others felt equipped to support them because of their own experiences. “*Okay with us teachers we know because we have daughters, we have gone through this so we know, so we just take it normal, talk to them and tell them that it is normal, it is healthy”* (P5 School 5, Baseline).

The way teachers communicate among themselves regarding girls’ menses can impact if students feel free to communicate with certain teachers. *“When they have problems they do not want other others to know, now when they approach teacher A and teacher A is a very harsh teacher and once you discuss that with her, she will discuss with others and the next thing the teacher will say “go away from me you are stinking” so that one you know will make the girl not to want her problems to be exposed”* (Px School 4, Baseline).

Some teachers noted that there is a difference in the role teachers can play based on their gender. This appears to be based both on student assumptions and teacher comfort. Some mentioned that students “*can go to any female teacher and explain, but they are very shy to come to male teachers”* (P1 School 3, Baseline). Male teachers also pass off this responsibility to female teachers, claiming that they have personal experience that can help these girls. *“It’s like also they undergo the same problem or they underwent the problem, they have experience on how they did it when they were young, it might not be appropriate for the male to handle the issues compared to the female teachers”* (P12 School 2, Baseline). When explaining why girls choose to and are directed to navigate school in gendered pathways as they do, one teacher related it to community tradition.

*“We cannot blame the male so much because it is like a tradition, because earlier on the girls were taught by their grandmother and boys by their grandfathers, so they believe that all that is in the life of girl should be in taught by a female and all that in the life of a boy should be taught by a man”* (P5 School 4, Baseline).

Several teachers recognized that it is important for male teachers to understand and be able to support girls during their menses. One female teacher specifically called out the promotion of this gender divide by exclaiming *“They should be able to handle; what if there is a school that does not have madam teacher will the girls suffer because there is no madam teacher!*” (P1 School 2, Baseline).

In Mid and Endline follow-up discussions, teachers at all schools noted that they saw how girls were more willing to tell them they were on their menses. *“Nowadays the girls are even telling teachers that they have that problem, like long time ago it was very difficult for them to tell you that she is experiencing her menses or to talk about it with us … but nowadays they take it as a normal thing to them*” (P1 School 4, Midline).

This impacted the gender divide as well, with teachers reporting that girls were more willing to talk to both male and female teachers about their menses. *“They know how to take care of themselves, and it has made most of the girls very free that when one fails, in that they need to go and change, they do not fear to approach male teachers, they will come and tell you teacher I am not feeling well because of abcd”* (Px School 2, Endline). They noted that there were still girls that were nervous to talk to male teachers however, with one teacher noting, *“they do not feel a hundred percent free with us, still they feel it is women affair, and so they feel that if they talk to us a lot about it, they do not feel confident”* (P6 School 5, Endline).

### 3.4. The impact of sensitization of menstruation

At baseline, teachers noted the need for girls to be sensitized as a means for improving MHH and academic performance.

*“You talk to them they do not want to respond they are moody throughout, so I think what you should do is to sensitize them, tell them the changes that may happen in their bodies when they are menstruating, assure them that menses are things that are very normal and advise them on what to do when they are menstruating”* (Px School 4, Baseline).

Teachers saw sensitization as a means for girls to understand their menses as normal. In their minds, early sensitization is a means for improving preparedness and wellbeing. *“We need to sensitize them that this is a natural occurrence that at certain period in your lifetime and at certain ages it will appear and when it appears it should not shock you”* (P5 School 1, Baseline).

Some teachers also noted that puberty education should occur sooner to achieve this goal, with one teacher stating, *“I think the basis of this topic about physical changes should be started in class four, because at class four, you can see some girls having those changes”* (Px School 4, Baseline).

As a result of sensitization through puberty education during the study interventions, most teachers noted perceived changes in the girls’ behavior. Specifically, they have noted that sensitization has contributed to a sense of normalcy about their menstrual cycle. They recognized that regarding their menses, *“they have taken it to be normal and in fact that they are just okay, so that’s why they have become very free, they play around with others being that they feel that they have protection”* (P9 School 6, Midline).

These improvements are not isolated to the girls in the study. Several teachers noted that:

*“Nowadays you find that those girls who are in the study educate those girls who are not in the study about the dates so you find that they know their dates exactly so that one minimizes their chances of going home because of the menstruation”* (P4 School 5, Midline).

This form of peer support recognizes the opportunities for girls to share what they learned in puberty education as a means of supporting their fellow classmates.

Many teachers also noted the need for boys to be sensitized as well as girls. *“I think what mwalimu [teacher] is speaking of is about sensitizing the boys also, you find that when the mooncups have been issued to the girls, they will hide it, and you know the boys will now start wondering”* (P5 School 5, Endline). At schools where boys and girls received puberty education together, boys were described as seeing menstruation as normal. *“We just talk to the boy’s, we tell them that those things are normal, any normal human being must undergo that. So we also tell them that their mothers also went through those stages, so they have taken it normal.”* (P10 School 4, Midline). This led to most teachers perceiving that girls feel more comfortable in the classroom, with one teacher stating, *“when a girl is on her period the girl will vacate from that desk and go and sit with the girls, but now the way they sit, they sit the same pattern as they always sit”* (P11 School 4, Midline).

Sensitization also plays a large role in the way that menstruation is perceived. As noted above, changing the view of menstruation as normal and not simply a problem to be resolved can improve outlook on menstrual issues. As one teacher noted:

*“How do you call nature a problem, because when I came in, madam number seven was talking about menstruation being a problem, but I do not think it is a problem, nature is not a problem, it is how we handle nature”* (P12 School 2, Midline).

## 4. Discussion

Understanding how teachers view their role in supporting schoolgirls’ MHH is essential for normalizing menstruation and improving the well-being of schoolgirls. These findings assist in understanding the short-term impact of puberty education and access to improved menstrual items on student performance and emotional wellbeing at the school. This study is one of a handful of studies that draws on both the male and female teacher perspectives on menstruation, and one of the few to focus exclusively on teacher perspectives based on the large body of data available. Previous studies were limited in their inclusion of teacher perspective, with a maximum of 14 teachers to be interviewed for the whole study (2, 4, 13, 15–17). Through our analysis of perspectives from 95 teachers, this study presents an opportunity to carefully examine the role teachers play in school environments.

While the teachers’ primary focus was girl’s academics, hygiene, and confidence, they also made a note of changes in dropouts and loosening of gender norms in communication with teachers from the baseline focus groups. Recognizing these changes is critical, as it informs how teachers perceive menstrual education, hygiene, and menstrual items in relation to improved management of menses. With this knowledge, it is important to navigate the roles that teachers currently play in girls’ management of their menses and the opportunities to enhance MHH through teacher and school-level involvement.

This study recognizes teachers as important actors in global MHH. Considering the significant amount of time that teachers spend with girls and the role they play in the community, their own knowledge and feelings towards menses can significantly influence girls’ emotions towards their own cycle. At baseline, many teachers discussed how some students avoided certain teachers known for sharing their private conversations or unnecessarily interrogating those that were feeling ill. Other were seen as trustworthy allies the girls could share with. Several teachers recognized their own practices and the need for them to adapt to ensure girls were comfortable communicating their menstrual issues. The support of teachers to understand the struggles of menstruating girls in schools, and also to normalize menstruation in their attitudes and responses, will help create a gender equitable environment in schools. (18). To do so, teachers themselves must be sensitized to ensure they can improve the support systems available to adolescent girls. In-service training of teachers, both male and female, has been recommended as a mechanism for their education on MHH (19, 20). The training of male teachers is critical given the high prevalence of male teachers in some countries, including Kenya (19). As our study demonstrated, male teachers will pass off menstruating students to female teachers given their ‘experience’ which can be problematic for those that do not have easy access to a female teacher. By encouraging in-service menstruation education for all teachers, they can serve as resources for their students and agents to help normalize the experience of menstruation, allowing girls to feel more comfortable in the classroom.

In addition to the sensitization of teachers, puberty education is a key piece of creating an empowering environment. Teachers discussed the potential for teaching school menstrual education sooner in addition to offering it for boys. These moves are supported by a 2016 UNICEF report, which calls for the early start to menstrual education (age 5–8) and the education of boys (20). It does so with the recognition that successful management can increase academic performance and proposes the implementation of a “MHH-supportive institutional environment” (20). By creating an environment that is conducive for girls to manage their menses as they need, schools are investing in their academic success. School-based menstrual health education is key, as it exposes girls to methods of management that parents may not be familiar with. As such, school-based menstrual health education as a form of formal sensitization is a tool for empowerment, ultimately enabling girls to effectively manage their own menstrual cycle. The Kenyan government recently approved Menstrual Hygiene Management education as a part of their government approved educational curriculum that addresses the menstrual needs of girls using a three-topic approach – Breaking the Silence, Safe and Hygienic Management and Safe Disposal (21).

Informal education fostered by the school is as critical to menstrual health education as formal education is. Teachers saw the impact of the study among girls who were not enrolled in addition to those that were. Girls were taught the tools they had learned to help them better manage their own menses by their classmates. Mason et al. (3) describes this phenomenon as the menstrual champion. Through peer support, girls were able to help sensitize their fellow classmates, thereby expanding the effects of the intervention (3). Our FGD suggests that these girls, known in the literature as ‘champions’ are effective at creating change through the sharing of this knowledge. Peer support groups, as an entity within the school, present an educational opportunity for girls to transmit educational information to each other. Recognizing this pathway is crucial as we look to develop further school-based interventions.

Through this study, it is recognized that an MHH-supportive environment cannot simply be one created by teachers’ behavior and puberty education, it is one that must be formed through the development of facilities that support the needs of the girls experiencing their menses. As discussed by Sommer and Sahin, “inadequate water and sanitation facilities pose a major impediment to school-going girls during menstruation” (22). While this issue was not prioritized in discussions in FGDs, some teachers noted that the washrooms in their school did not afford girls the privacy they needed, resulting in them going home to change their menstrual products. While certain facilities were equipped with full washrooms for the girls, others were not. The nature of the MS study was that each school was chosen based on different levels of adherence with WASH protocols, so the noted differences between facilities is in line with the study design. There was not a strongly recognized association between the school facilities and girls’ behavior. Information obtained in focus groups conducted with girls in the MS Study suggests otherwise. Oduor et al. (23) identifies the provision of latrines in these schools as responsible for the delaying of changing menstrual products, or ‘over-staying’ as the girls described. Lack of locks, little light, and lack of space, in addition to school breaks not being long enough were cited as clear infrastructural and institutional factors that hindered girls’ ability to change their menstrual products properly (23). As we examine behavior in relation to menses, it is critical that each school examines the built environment around these girls, including the infrastructure and schedule as it impacts girls’ behavior in their management of menses. In order to build an MHH-supportive environment, both the adequacy of the facilities in addition to sensitization must be considered to have a significant and sustainable impact.

As we examine next steps for improving MHH championing schools, it is crucial to recognize the individual, culturally tied experience of menses that girls experience. Incorporating this understanding of menstruation in schools through sensitization of teachers, puberty education, and infrastructural changes will only further empower girls to manage it as best fits in their own cultural context.

### 4.1. Limitations

A key limitation of the study is that not all teachers participated in the three FGDs for their school, with some only participating in one or two. However, while this prevented some teachers from commenting critically on students before, during, and after the interventions, many who were new to the focus groups were able to draw upon their previous experience which informed their opinions.

While we have knowledge of the fact that both male and female teachers participated in each of the FDGs, gender was not recorded in the transcripts to maintain the confidentiality of the participants, thus we do not have a ratio of the male to female teacher participation per FGD and were unable to conduct further analysis on differences in opinion by gender. While gendered language was sometimes used in the FDGs, allowing some conclusions to be made about how students approached teachers, the lack of availability of this information makes it difficult to make wider assessments of gendered expectations of teachers in addressing menstruation in schools.

In addition, this analysis is based on results from the MS study in the year following intervention. There is no information on whether this study had a longer-term impact on teachers or the effect on school culture around menstruation. The study authors also recognize that the data was collected 10 years ago and that current advancements may impact the utility of the results. Study findings and study researchers helped to inform limited progress over the past decade in guidelines for teachers as well as establishing MHH strategies and policies, including the Ministry of Health Menstrual Hygiene Management strategy and Menstrual Hygiene Management in Schools: A Handbook for Teachers (21, 24). Additionally, a program that supplied sanitary products to some of the most vulnerable girls in Kenya was developed and implemented in 2017 by the State Department for Gender and Affirmative Action, although this program was not informed by the study findings. While these changes are important, they are limited in scope and impact. Thus, the analysis presented here remains significant despite the years that have passed since the research team collected the data used in this analysis.

## 5. Conclusion

This study illustrates the important role that teachers play in MHH. The results of this study build on existing MHH research that recognizes the necessity of building an MHH-supportive environment in schools. Through its approach of incorporating teacher views, it offers a new perspective in the understanding of MHH interventions at the school level. Teacher knowledge, existing opinions of MHH, and thoughts on the types of interventions present an opportunity to incorporate their understanding of the barriers to MHH and cultural attitudes at the school as a whole that would benefit future MHH interventions, and aid in adapting the theoretical concept of the MHH-supportive environment into practice at their schools.

## Data availability statement

The datasets presented in this article are not readily available because this study was conducted with approval from the Kenya Medical Research Institute (KEMRI) Scientific and Ethics Review Unit (SERU) which requires that data should be released from any KEMRI-based Kenyan study (including deidentified data) only after written approval for additional analyzes. In accordance, data for this study will be available upon request, after obtaining written approval for the proposed analysis from the KEMRI SERU. Their application forms and guidelines can be accessed at https://www.kemri.org/seru-overview. Requests to access the datasets should be directed to KEMRI SERU, seru@kemri.org.

## Ethics statement

The studies involving human participants were reviewed and approved by Kenya Medical Research Institute (KEMRI) Scientific and Ethics Review Unit (SERU). The patients/participants provided their written informed consent to participate in this study.

## Author contributions

PP-H, LM, and KA conceived and designed the study. LM, EN, KO, and PP-H performed the experiment. JS and LN performed the qualitative analysis. JS wrote the first draft of the manuscript. All authors contributed to the article and approved the submitted version.

## Funding

This study was funded by the UK Medical Research Council (MRC)/Foreign, Commonwealth and Development Office(FCDO)/Wellcome grant (G1100677/1). The funders had no role in study design, data collection and analysis, decision to publish, or preparation of the manuscript.

## Acknowledgments

The authors thank the Head Teachers and school staff, parents, and children in schools in Gem sub-County for their enthusiasm to participate in this study. Field and office staff are thanked for their hard work and diligence. The Director of KEMRI has approved this manuscript.

## Conflict of interest

KA was employed by Care International.

The remaining authors declare that the research was conducted in the absence of any commercial or financial relationships that could be construed as a potential conflict of interest.

## Publisher’s note

All claims expressed in this article are solely those of the authors and do not necessarily represent those of their affiliated organizations, or those of the publisher, the editors and the reviewers. Any product that may be evaluated in this article, or claim that may be made by its manufacturer, is not guaranteed or endorsed by the publisher.
